# Efficient Detection of Malicious Traffic Using a Decision Tree-Based Proximal Policy Optimisation Algorithm: A Deep Reinforcement Learning Malicious Traffic Detection Model Incorporating Entropy

**DOI:** 10.3390/e26080648

**Published:** 2024-07-30

**Authors:** Yuntao Zhao, Deao Ma, Wei Liu

**Affiliations:** School of Information Science and Engineering, Shenyang Ligong University, Shenyang 110159, China; zhaoyuntao2014@163.com (Y.Z.); deaoma_9810@163.com (D.M.)

**Keywords:** network security, deep reinforcement learning, entropy, decision tree proximal policy optimisation, malicious traffic detection

## Abstract

With the popularity of the Internet and the increase in the level of information technology, cyber attacks have become an increasingly serious problem. They pose a great threat to the security of individuals, enterprises, and the state. This has made network intrusion detection technology critically important. In this paper, a malicious traffic detection model is constructed based on a decision tree classifier of entropy and a proximal policy optimisation algorithm (PPO) of deep reinforcement learning. Firstly, the decision tree idea in machine learning is used to make a preliminary classification judgement on the dataset based on the information entropy. The importance score of each feature in the classification work is calculated and the features with lower contributions are removed. Then, it is handed over to the PPO algorithm model for detection. An entropy regularity term is introduced in the process of the PPO algorithm update. Finally, the deep reinforcement learning algorithm is used to continuously train and update the parameters during the detection process, and finally, the detection model with higher accuracy is obtained. Experiments show that the binary classification accuracy of the malicious traffic detection model based on the deep reinforcement learning PPO algorithm can reach 99.17% under the CIC-IDS2017 dataset used in this paper.

## 1. Introduction

In today’s digitised world, cybersecurity faces increasingly complex and unpredictable threats, and malicious network traffic is a significant issue in the field of cybersecurity. Malicious traffic refers to those data streams that attempt to violate, damage, or steal information from network systems, and the dangers not only involve the privacy and security of businesses and individuals but also have a substantial negative impact on the entire network ecosystem. According to Netscout’s H1 2023 DDoS Threat Intelligence Report, the maximum DDoS attack bandwidth in H1 2023 grew to 978.5 Gbps compared to H2 2022, with over 7.85 million attacks worldwide. This shows that malicious traffic remains a prevalent and severe cybersecurity threat facing Internet users today.

Malicious traffic may manifest in various forms, including malware distribution, network intrusion, denial-of-service attacks, etc. These attacks may lead to data leakage and service disruption and may even be used to commit more insidious cybercrime activities. According to the Crypto Attack Landscape Report 2023, 85.9% of cyber threats are now initiated through encrypted channels, with browser vulnerabilities and ad spyware sites increasing by 297.1% and 290.5% each year, respectively. This reinforces the need for security operators to thoroughly examine all network traffic. In the face of these potential threats, it has become imperative to protect the network from malicious traffic.

In response to evolving cyber threats, researchers and security experts have been striving to improve the efficiency of malicious traffic detection. Traditional detection methods mainly rely on a manual design based on rule and feature engineering. However, with the development of deep learning techniques, deep reinforcement learning shows strong potential in malicious traffic detection.

Deep reinforcement learning adapts better to the changing patterns and dynamic behaviours of new threats by learning from network traffic’s complex patterns. It simulates an agent’s behaviour in a complex environment and continuously optimizes its decision-making strategy through reward and punishment mechanisms. Deep reinforcement learning introduces a new approach to malicious traffic detection. Building upon the machine learning decision tree algorithm and the proximal policy optimisation (PPO) algorithm in deep reinforcement learning, this paper incorporates the concept of information entropy to propose an Efficient Detection of Malicious Traffic using a Decision Tree-based PPO (EDT-PPO) model, achieving the efficient detection of malicious traffic.

The main contributions of this paper are as follows:

1. Initial classification predictions were made for the dataset using decision tree principles. Individual decision trees used information entropy as a criterion for feature splitting when performing feature splitting. The importance score of each feature was obtained by calculating the information gained from the feature during the classification process. Information entropy measures the uncertainty of the data. The lower the information entropy of the features, the faster the deep reinforcement learning model can learn the true pattern of the data and adapt to different data distributions and task requirements, thus speeding up the convergence of training and improving the flexibility and robustness of the model. The optimal number of features was selected by comparing the accuracy of subsequent classification with the different number of features. Thus, the performance of the model is optimised. Experiments show that after feature selection, the accuracy of the dataset is improved by 3.08% under the deep reinforcement learning model.
2. Build a new malicious traffic detection model using a proximal policy optimisation algorithm in deep reinforcement learning. The algorithm uses an Actor-Critic structure to detect network attacks. A truncation factor is set to limit the network from making substantial updates during the process of updating the network parameters. The stability of the network parameter update is increased to a great extent while ensuring the accuracy of the network. Through experimental comparison, the accuracy and F1 score of the PPO detection model is improved by 0.94% and 0.93%, respectively, compared to the existing LSTM detection model and CNN detection model. The accuracy and F1 score were improved by 0.74% and 0.69%, respectively, compared to the better-performing DQN model.
3. In the optimisation process of the PPO algorithm, an entropy regularity term is introduced in the loss function. The entropy regularity term will encourage the strategy to perform more exploration in situations with high uncertainty. This helps the algorithm to better explore the environment and learn more valid information, thus improving the efficiency and performance of training. To compensate for the lower accuracy of the model due to a lot of exploration in the early stages of training, we introduce an attention mechanism in both neural network parts of the model. This allows the model to focus more on the learning of important features while suppressing the over-learning of minor features, thus improving the model’s ability to represent and generalise the data.

The remainder of this paper is organized as follows: In Section 2, We present the development process of malicious traffic detection methods and the related work. In Section 3, we detail the process of building the PPO malicious traffic detection model. Section 4 illustrates the performance of the constructed model by analysing the accuracy, convergence speed, and stability of the algorithm. Simulation, numerical results and analysis are also presented. The limitations of the current study and potential avenues for future research are addressed in Section 5. Section 6 concludes the paper.

## 2. Related Work

The initial approach to detecting malicious traffic was based on statistical learning. Historical traffic data is used and analysed using statistical methods. Statistical features are extracted and a mathematical model is constructed. Subsequently, the model is applied to new network data traffic and analysed for malicious traffic detection. Ref. [1] proposes an intrusion detection system based on statistical analysis using machine learning methods. The system enables the software-defined network controller to detect malicious traffic and avoid potential losses such as system failure or risk of attack; Ref. [2] extracts and trains statistical features from the content of HTTP POST requests for benign and malicious traffic tracking. A low false positive rate is achieved; Ref. [3] proposes a time-sequence-based technique for anomalous traffic detection. The method utilises time interval timers and time counters to monitor the sequence and arrival intervals of traffic. Thus, the anomalous traffic is labelled. Statistical learning-based network anomalous traffic detection techniques are not only able to effectively deal with dynamic changes in information systems. They can also identify the abnormal behaviour of the system by learning the probability distribution. However, this method is relatively mature and requires less computer performance. However, once abused by attackers, it may lead to the problems of large errors and high false alarm rates.

In recent years, with the rapid development of machine learning (ML) [4,5], various machine learning algorithms have also been applied to malicious traffic detection. Ref. [6] proposed an intrusion detection system combining an improved genetic algorithm and K-means algorithm, which obtained better detection efficiency. Ref. [7] advocates the use of principal component analysis for anomalous traffic detection. The detection efficiency is improved by analysing the shrinkage ratio, the effect of noise, and the number of desirable principal components. Ref. [8] reduces the dimensionality of input features based on feature vitality and utilises the Naive Bayes algorithm for intrusion detection. The selected features significantly improve the detection performance. Ref. [9] used the J48 decision tree algorithm to construct an intrusion detection model. The performance of the model is validated on the Kyoto 2006+ dataset and high detection accuracy is achieved. Ref. [10], on the other hand, improves the efficiency of detection by using Bayesian networks and feature selection; Ref. [11] introduces an agglomerative hierarchical clustering algorithm capable of detecting anomalies in mixed datasets containing numerical and other categorical attributes; Ref. [12] combines the Tree Seed Algorithm TSA and K Nearest Neighbour Algorithm to construct an intrusion detection model that can effectively remove redundant features; Ref. [13] improves the active learning multi-class support vector machine algorithm and proposes a cost-sensitive support vector machine, CMSVW. Experiments show that the CMSVW algorithm achieves some improvement on the data imbalance problem. Machine learning has greatly promoted the development of cyber security technology, but still suffers from ground accuracy and a high false alarm rate. Moreover, machine learning algorithms rely heavily on a priori knowledge, which leads to the loss of the generalisation ability and robustness of machine learning algorithm models.

The rapid development of computer technology and the rise of deep learning gradually provide a new idea for network abnormal traffic detection. Ref. [14] proposed a network traffic detection model based on a Deep Belief Network (DBN) using the theory of deep learning, which solves the problem that traditional neural networks tend to fall into a local optimum. Ref. [15] concludes by experimentally comparing classical models in deep learning that a convolutional neural network shows good performance in traffic detection. Ref. [16] employs convolutional neural networks (CNNs) and Long Short-Term Memory (LSTM) networks for feature learning. Experimental results demonstrate the model’s effectiveness in reducing the false alarm rate (FAR). Ref. [17] proposes a deep learning-based DDoS attack detection method that utilizes recurrent deep learning networks to learn traffic sequences of network attacks. Compared to traditional machine learning methods, it demonstrates greater advantages in reducing false alarm rates. Ref. [18] employs a method of converting network traffic into images and utilizes convolutional neural networks (CNNs) for recognition and detection. The final results indicate that although this method can be applied to intrusion detection, it does not entirely improve upon existing techniques. Ref. [19] proposes the use of Recurrent Neural Networks (RNNs) for network intrusion detection. Comparisons with traditional machine learning algorithms demonstrate that their detection performance surpasses that of traditional classification methods, providing a new research approach for intrusion detection. Ref. [20] proposes a new approach. The raw traffic is segmented based on sessions and transformed into images to achieve end-to-end detection. Although it avoids feature design and extraction, it suffers from the problem of running too slow. Ref. [21] introduced the theory of deep learning to detect malicious traffic using the idea of combining raw data and feature engineering. A deep neural network model combining multilayer perceptron and a convolutional neural network is proposed. The data were pre-processed with tensorization. The results show a large improvement in detection accuracy and the time cost compared to traditional machine learning methods such as SVMs and decision trees. However, this model ignores the temporal and correlation nature of malicious behaviours and it is weak in detecting hidden malicious behaviours. Ref. [22] proposes an end-to-end one-shot detection method. By converting sample data into grayscale images and using neural networks to train and learn from these image samples, malicious traffic is detected through a grayscale comparison between samples. This method also partially addresses the issue of data sample imbalance. However, its performance in detecting new types of attacks is unsatisfactory, requiring frequent updates to the sample library to maintain a high detection rate.

Although deep learning has a great advantage in feature learning and extraction, it is highly dependent on the data used in the training process. A large amount of labelled data is required to train the model. Reinforcement learning (RL), on the other hand, can reduce the model’s dependence on data by using unlabelled data and pursuing the method of maximising rewards by obtaining feedback through interaction with the environment. In the field of cyber security, researchers have conducted many studies on reinforcement learning applied to intrusion detection. Ref. [23] concludes by studying the application of reinforcement learning in intrusion detection. Although reinforcement learning has not achieved the same performance as supervised machine learning algorithms, it has demonstrated certain advantages in terms of dynamics. Ref. [24] combines reinforcement learning with multiple resampling algorithms to create an adaptive learning environment. It integrates the True False Rate Synthetic Minority Oversampling Technique (TFRSMOTE) algorithm to achieve high detection accuracy on the NSL-KDD dataset. Ref. [25] combines reinforcement learning with evolutionary learning to propose an adaptive database intrusion detection model. By using the interactive updates of a behaviour network and an evaluation network, the model achieves intrusion detection. Experimental results show that this model can adaptively learn intrusion behaviours, thereby improving detection performance. Although reinforcement learning has shown better results in intrusion detection, it still has some limitations when facing problems with high-dimensional features, so researchers have started to combine deep learning and reinforcement learning to solve such problems.

Deep reinforcement learning (DRL) has had notable success in gaming and robotics, and as a result, researchers have begun to expand Deep reinforcement learning into cybersecurity. Ref. [26] builds an adaptive cloud intrusion detection system based on deep reinforcement learning. The experimental results show that this system can detect new types of attacks and still maintains a low false positive rate with improved accuracy. The authors of the Ref. [27] proposed a DRL-based intrusion detection system with self-updating capability. It can handle millions of network traffic data in real time. Ref. [28] applied DQN, DDQN, policy gradient and AC algorithms for network intrusion detection. Through comparison, it is found that DDQN has the best results. Ref. [29] developed an environmentally adversarial deep reinforcement learning detection framework. The model is trained by generating new traffic samples by simulating the environment. Although the detection of a few classes of samples is better, the time cost is significantly higher than that of a general deep reinforcement learning model. Ref. [30] proposes a deep reinforcement learning framework for anomaly detection in Supervisory Control and Data Acquisition (SCADA) systems. The model uses a ‘Q-network’ that achieves state-of-the-art performance in pattern recognition for complex tasks. With the continuous enhancement of computer device performance, the future development of deep reinforcement learning will trend toward structural diversity and complexity.

In this paper, based on existing research, the deep reinforcement learning proximal policy optimisation algorithm is introduced to the field of network security, combined with the feature selection of a decision tree algorithm, which overcomes the limitation of reinforcement learning on high-dimensional data, and builds a more stable and efficient malicious traffic detection model.

## 3. Malicious Traffic Detection Model Design

### 3.1. Dataset Selection and Pre-Processing

The CIC-IDS2017 (Canadian Institute for Cybersecurity Intrusion Detection System 2017 Dataset) is a dataset for network intrusion detection research created by the Canadian Institute for Cybersecurity. The dataset collects a large amount of network traffic data, including normal traffic and many types of malicious traffic, so researchers and practitioners can use it to evaluate and develop intrusion detection systems. The CIC-IDS2017 dataset is collected from real network traffic, which ensures its high authenticity and realism. This enables the dataset to better reflect the situation in the actual network environment and increases the study’s credibility. The dataset consists of 2,830,743 pieces of data with 79 dimensional features. The “Label” identifies the sample’s specific type of malicious behaviour, which is extracted and saved separately for subsequent use. The exact data types of the dataset are shown in Table 1 below.

Not all the samples in the dataset can be used. There are NAN and INF values in some of the sample features and such samples cannot be recognised using the detection model. So, such useless sample data need to be removed. The search of the procedure revealed the presence of some features whose values were all zero. Such features could not play any role in performing network traffic identification, so such features were also removed. The specific feature names are shown in Table 2 below.

Among the remaining data sample features, there are some features with high correlation. In pre-processing the dataset, removing features with high correlation avoids multicollinearity, reduces the complexity of the model, and improves the interpretability and generalisation of the model. In this paper, we filter the features with high correlation by calculating the correlation matrix of all the features. By calculating the correlation, 25 highly correlated features are finally filtered out and their deletion operation is performed. These features are listed in Table 3.

**Figure 1:**
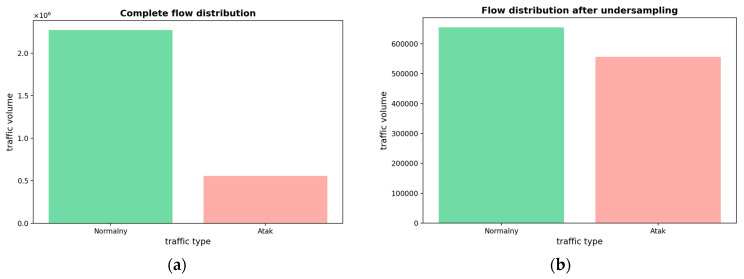
(**a**) Flow distribution before random undersampling; (**b**) flow distribution after random undersampling.

Since the dataset is unbalanced, an imbalance in the proportion of normal traffic and cyber attack traffic will result in the model being more inclined towards the identification of majority class samples. So, a random undersampling technique is used to process the dataset to balance the ratio of normal and abnormal traffic in the dataset to obtain data samples that can be used for the model to perform training. The flow distribution before and after random undersampling is shown in Figure 1.

Subsequently, the numerical features of the dataset are normalised and scaled to be between 0 and 1 for the model to be trained. The dataset was divided with 70% going into a training set and 30% going into a test set, where the training set was used to train the model and the test set was used to validate the performance of the model. Although a random undersampling operation is performed on the dataset, there are still a few classes of samples in the dataset. For example, the penetration attack class has only 24 sample flows. In order to improve the model’s recognition rate of such traffic and further reduce the gap between the proportion of normal and abnormal traffic, a random oversampling operation is performed on the training set. The test set is not subjected to oversampling operations; the reason for this is to ensure that the traffic samples in the test set are more closely aligned with the actual network traffic, making the test results of the model more convincing.

After the above process, the resulting dataset will be predicted using the decision tree algorithm for initial classification, and the features’ importance scores will be calculated.

### 3.2. Feature Selection

For the CIC-IDS-2017 dataset, the entropy-based decision tree classifier has several advantages. Firstly, this dataset contains multiple types of network traffic features, there may be complex non-linear relationships between these features, and the decision tree can effectively capture these complex relationships and filter out the most discriminative features, thus improving the performance of the classifier. In addition, the decision tree calculates the information gained from each feature during the construction process and ranks the features according to their importance, allowing us to intuitively understand which features contribute the most to the classification results. Decision trees can also handle multi-class classification problems with a degree of robustness and ease of interpretation, which is useful when analysing network traffic attacks. In contrast, filter methods usually evaluate each feature independently without considering the dependencies between features; wrapper methods require multiple trainings of the model, have a high computational overhead, and depend on specific learning algorithms; and decision trees as an embedded method are more efficient as they perform feature selection directly during the training process without additional steps.

A decision tree [31] is a commonly used machine learning algorithm that recursively partitions a dataset to construct a tree-like structure for classification or regression. In the process of building a decision tree, information entropy is an important concept used to measure the purity or uncertainty of the data. Information entropy is a concept introduced by information theory to estimate the uncertainty of a random variable. In decision trees, we use information entropy to evaluate the importance of each feature in order to select the optimal feature for node partitioning. The formula for calculating information entropy is as follows:

(1) $$H\left(X\right)=-\sum_{i=1}^{n}\;P\left(X_{i}\right)\log_{2}P\left(X_{i}\right)$$

where is the information entropy of the random variable *X*, *P*(*x_i_*) is the probability that the random variable *X* takes the value *x_i_*, and *n* is the number of values of the random variable *X*.

In the process of decision tree construction, we first calculate the information entropy of each feature and then select the optimal feature for node division according to the information entropy. Specifically, we partition each value of each feature, calculate the information entropy of the subset after partitioning, and select the feature and value that minimises the information entropy as the partitioning feature of the current node. This process is carried out recursively until the number of samples in the node falls below the threshold we set.

**Figure 2:**
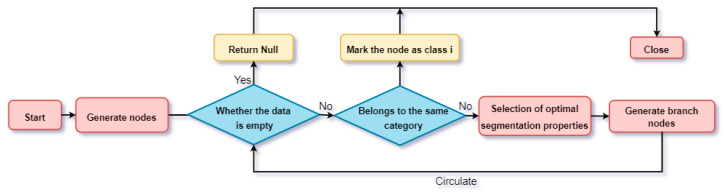
Diagram of the decision tree construction process.

The specific process of the decision tree classifier is as follows. Firstly, using the training dataset obtained in Section 3.1 as the sample set *S*, while the sample set is continuously split to generate the malicious traffic feature decision tree, the attribute with the slightest current value is selected as the split node by calculating the information entropy of each feature attribute. Repeatedly, the sample set can be hashed into subsets according to this criterion. Suppose the tuple categories contained within the ith sample subset are the same during the sample set splitting process. In that case, the current node can be regarded as the leaf node of the split decision tree at this point, and the split is terminated. Suppose a subset of malicious traffic attributes is generated during the decision tree splitting process that does not satisfy the above conditions. In that case, the decision tree continues to be generated recursively using the above methodology in turn until all the malicious traffic subsets contain tuples belonging to the same class. In constructing the decision tree, the information entropy reduction of each feature is calculated and normalised to obtain the degree of contribution of each feature in the classification process, i.e., the feature importance score. The decision tree construction process is shown in Figure 2.

A reasonable threshold is set based on the number of features required; features less than this threshold are filtered based on the importance score and removed using the drop function. The remaining features are the ones that have a high contribution in distinguishing between normal and abnormal traffic, and they are more beneficial for the model’s training.

**Figure 3:**
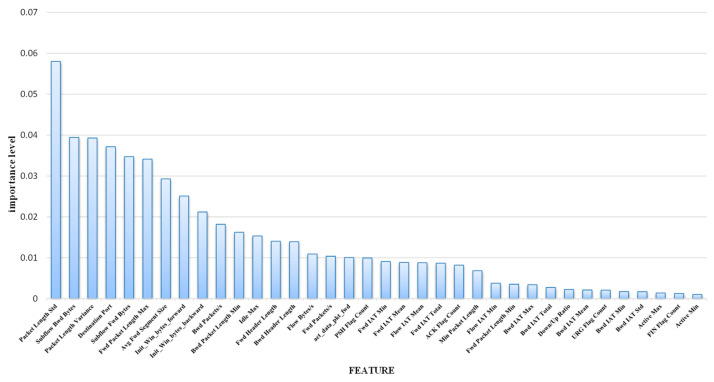
Importance of features.

The processed dataset in Section 3.1 is predicted by the entropy-based decision tree classifier for initial classification to obtain the ranking of the contribution of each feature in the classification process. The features with contributions less than 0.001 are deleted, and finally, the top 36 remaining features with higher importance scores are shown in the following Figure 3.

**Figure 4:**
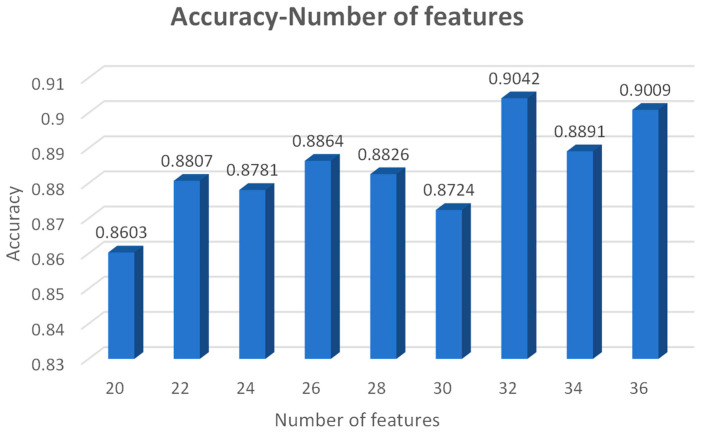
Accuracy–Number of features.

By choosing the different number of features and handing over the dataset to the model for classification and prediction, the histogram, as shown in Figure 4, was obtained. It was observed that the highest accuracy was achieved in classifying the dataset using 32 features, so all the subsequent experiments were conducted using the 32 features selected in this section for the training and testing of the model. The number of the features in the graph indicates the most contributing features, e.g., a feature number of 20 indicates that the selected features ranked in the top 20 in terms of importance score. 

### 3.3. PPO Detection Model

#### 3.3.1. Natural Policy Gradient Algorithm

The natural policy gradient algorithm exposes the shortcomings of traditional policy gradient algorithms and ways to remedy them. Although the natural gradient has surpassed algorithms such as TRPO and PPO, its fundamentals are crucial to contemporary RL algorithms.

In traditional policy gradient algorithms, we update policy weights based on the gradient of the objective function and the step size. However, this updating process may encounter two common problems:

1. Overshooting: the update misses the reward peak and falls into the suboptimal strategy area.
2. Undershooting: taking an update step that is too small in the gradient direction can lead to slow convergence.

In supervised learning problems, overshooting does not cause significant issues because the data is static, and corrections can be made in the following training iteration. However, in reinforcement learning problems, overshooting may result in getting stuck in a poor policy region, and future batches of data samples may not provide helpful information. Updating the policy with poor-quality data samples can lead to negative feedback loops that are difficult to recover from. A lower learning rate can partially alleviate this issue but may result in slower convergence rates, leading to undershooting problems. To avoid the severe consequences of overshooting, a straightforward approach is to limit the upper limit of each update step:

(2) $$\Delta \theta^{*}=\underset{\left|\Delta \theta \right|\leq \epsilon }{\mathrm{argmax}}J\left(\theta +\Delta \theta \right)$$

where ∥Δθ∥ represents the Euclidean distance of the strategy weights before and after the update. Δθ^*^ denotes the optimal parameter increment found based on the current parameter *θ*.ϵ denotes the value of the parameter increment’s paradigm constraints.

The final result differs from traditional policy gradient algorithms in two aspects: The first consideration is the sensitivity of the strategy to local variation, with the strategy gradient corrected by the inverse Fisher matrix, whereas traditional gradient methods assume updating to the Euclidean distance. The second is that the update step has a dynamic expression that adapts to the gradient and local sensitivity, ensuring that the magnitude of the policy change is a fixed value regardless of the parameterisation. In traditional methods, the update step is usually set to some standard value, such as 0.1 or 0.01.

#### 3.3.2. Trust Region Policy Optimisation Algorithm

The Trust Region Policy Optimisation (TRPO) algorithm [32] is the foundation of modern reinforcement learning, which is based on natural policy gradient optimisation and quickly gained popularity as a mainstream reinforcement learning algorithm because it empirically performs better and more consistently than natural policy gradient algorithms. Although it has since been surpassed by proximal policy optimisation (PPO), it is still important. To address the problem of natural strategy gradient algorithms, we would like to quantify the optimisation of the strategy to ensure that each update is optimally functional. We need to calculate the difference in expected returns between the two strategies to do this. The approach adds the original strategy’s anticipated return to the new strategy’s expected advantage. This expression computes the dominance function under the original strategy without resampling:

(3) $$J\left(\pi_{\theta +\Delta \theta }\right)=J\left(\pi_{\theta }\right)+E_{\tau ∼\pi_{\theta +\Delta \theta }}\sum_{t=0}^{\infty }\;\gamma^{t}A^{\pi_{\theta }}\left(s_{t},a_{t}\right)$$

where the dominance function is defined as

(4) $$A^{\pi_{\theta }}\left(s,a\right)=E\left(Q^{\pi_{\theta }}\left(s,a\right)-V^{\pi_{\theta }}\left(s\right)\right)$$

Here, *s* represents the state, *a* represents the action, and *π_θ_* represents the policy with parameter *θ*, describing the probability distribution of taking each action in each state. E represents the expectation of a random variable. *J*(*π_θ_*) represents the objective function value under the current policy. $A^{\pi_{\theta }}\left(s,a\right)$ represents the advantage of taking action *a* in state *s* under the current policy relative to the average level. $Q^{\pi_{\theta }}\left(s,a\right)$ represents the expected cumulative reward for taking action *a* in state *s* and subsequently following the policy. $\left.V^{\pi_{\theta }}(s\right)$ represents the expected cumulative reward for following policy *π_θ_* in state *s*.

Computing the inverse Fisher matrix in natural policy gradient algorithms is time-consuming and numerically unstable, especially for neural networks where the parameter matrix can become very large. The conjugate gradient method is introduced, a numerical process that approximates the product of the above equation to avoid computing the inverse matrix. Conjugate gradients typically converge within $\left|\theta \right|$ steps, thus allowing large matrices to be handled. TRPO performs conjugate gradient algorithms, line searches for constrained sample KL dispersion, and checks for improved substitution advantages. It represents an essential milestone in developing natural strategy gradient algorithms by providing a significant improvement compared to natural strategy gradient algorithms.

#### 3.3.3. Proximal Policy Optimisation Algorithm

Proximal policy optimisation (PPO) is a highly regarded algorithm in the field of reinforcement learning that aims to solve the problem of optimising policies to maximise cumulative rewards. The development of PPO can be traced back to 2017 [33], when researchers at OpenAI proposed it, and it has quickly become a hot topic in the reinforcement learning community. There are several important reasons behind its success. Firstly, PPO is a proximal policy approach designed to address the trade-off between stability and performance in policy optimisation problems. It introduces a concept of truncated trust domains that limit the size of policy updates to ensure training stability. This mechanism allows PPO to excel in coping with problems in continuous action spaces and high-dimensional state spaces. The core idea of PPO is to improve the policy during policy iteration by maximising the expected return. However, to prevent the policy from changing too drastically, PPO introduces a clipped surrogate objective to limit the size of each policy update. This trust region is an important mechanism that balances strategy improvement and stability, enabling PPO to achieve stable and efficient policy optimisation without complex hyperparameter tuning. Successful applications of PPO cover a variety of fields, including robot control, autonomous driving, game playing, and financial trading. In robot control, PPO is used to train robots to perform various tasks such as grasping objects, walking and navigating. In autonomous driving, PPO can train self-driving cars to cope with complex traffic environments. In game playing, PPO has achieved significant success in defeating human champions in Go and Texas Hold’em tournaments. In addition, PPO is used in the financial sector to develop automated trading systems.

The PPO algorithm uses two neural network models, the central role of which is to judge the state characteristics of the current input and determine whether the traffic is an abnormal flow. The two networks are the Actor network and the Critic network, where the input to the Actor network is the state of the current environment, and the output is the probability of action. The Actor defines which actions should be taken to maximise the cumulative reward in a given state. It takes the current environmental state as input and outputs the probability of each possible action. The input to the Critic network is the current state, and the output is the value of the current state, which measures the expected cumulative reward that an intelligent body can obtain by following the current strategy from this moment onwards in the current state. In addition to defining the two networks, the experience repository pool TRANSACTION for the PPO algorithm is defined to hold the parameters obtained for each trajectory. Unlike the DQN algorithm, the purpose of the experience replay buffer is to facilitate the computation of the cumulative discounted return and advantage for each trajectory rather than to eliminate the correlation between experiences.

Because the Actor network needs to output as much action advantage as possible, the advantage function is defined to evaluate the effectiveness of the action, and the advantage function is defined as follows:

(5) $${\hat{A}}_{t}=\delta_{t}+\left(\gamma \lambda \right)\delta_{t+1}+\cdots +{\left(\gamma \lambda \right)}^{T-t+1}\delta_{T-1}\;\mathrm{where}\;\delta_{t}=r_{t}+\gamma V\left(s_{t+1}\right)-V\left(s_{t}\right)$$

where is the return received in the current state, $V\left(s_{t}\right)$ is the value of the current state, which is arrived at by the Critic network based on the value of the current state, γ and λ are discount factors, which are used to measure how much the future return affects the current state. ${\hat{A}}_{t}$ represents the estimated advantage function at the time step *t*. $\delta_{t}$ represents the temporal difference error at the time step *t*, measuring the difference between the predicted value and the actual reward plus the discounted value of the next state.

According to the dominance function, the Actor network loss function of the PPO algorithm is defined as

(6) $$L^{CLIP}\left(\theta \right)={\hat{E}}_{t}\left[\min\left(r_{t}\left(\theta \right){\hat{A}}_{t},\mathrm{clip}\left(r_{t}\left(\theta \right),1-\epsilon ,1+\epsilon \right){\hat{A}}_{t}\right)\right]\;\mathrm{where}\;r_{t}\left(\theta \right)=\frac{\pi_{\theta }(a_{t}|s_{t})}{\pi_{\theta_{\mathrm{old}}}(a_{t}|s_{t})}$$

Here, *s* represents the state, *a* represents the action, *r* represents the immediate reward, *θ* represents the parameters of the neural network at each iteration, and ϵ is a hyperparameter that can be used to limit the extent of each gradient update, ensuring that the network does not miss the optimal parameters due to overly large updates, and that the network converges more stably.

After updating the Actor network using the above loss function, data from the experience replay buffer is retrieved to update the Critic network. The computed discounted return is subtracted from the current state value predicted by the Critic network, and the mean squared error (MSE) function is used as the loss function for the Critic network for training. After that, the parameters of the two neural networks are continuously updated to make the output probability of the correct action as large as possible, using gradient descent to update their parameters until the neural networks converge.

The detection of malicious traffic using the PPO algorithm consists of three main steps; firstly, the model is trained using a large amount of traffic sample data so that the model can identify the characteristics of network traffic; then, the parameters of the neural network in the model are updated using a large amount of data from the experience pool; finally, the trained model is used to detect abnormal traffic.

To implement the aforementioned steps, we use the PyTorch framework to construct the neural network models for the Actor and Critic networks. We adopt a three-layer fully connected structure, with ReLU [34] activation functions in the first two layers. The output of the final layer of the Actor network corresponds to the dimensions of the action space, while the final fully connected layer of the Critic network has a dimension of 1, outputting the value of the current action. In addition, in order to improve the ability to enhance the exploration of the PPO algorithm in the pre-training period, an entropy regularisation term is introduced into the loss function of the Actor, which increases the exploration of the strategy by maximising the entropy of the strategy. In this paper, the strategy entropy is calculated as follows:

(7) $$H=-\sum_{i=1}^{2}\;p_{i}\log\left(p_{i}\right)$$

where *p_i_* represents the probability of each action output by the policy function. When the model is sufficiently converged, a entropy regularisation term with a high weight may bring negative effects to the model. Therefore, the weight of the entropy regularisation term will be slowly reduced with training to ensure that the model can converge to a better state. In order to compensate for problems such as low accuracy due to extensive exploration during the pre-training phase of the model, an attention mechanism is added to the two neural network parts of the model, which allows the model to pay more attention to important features and suppress unimportant features, thus improving the model’s ability to characterise and generalise the data.

In this paper’s malicious traffic detection model, the model needs to update the parameters using the reward values obtained by the intelligence each time. The reward values for the PPO detection model are defined as follows:

(8) $$r=\left\{\begin{array}{ccc} 10, & & True\\ 0, & & False \end{array}\right.$$

When the agent makes a correct decision, it receives positive feedback of 10 points; otherwise, making an incorrect decision results in negative feedback of 0 points.

**Figure 5:**
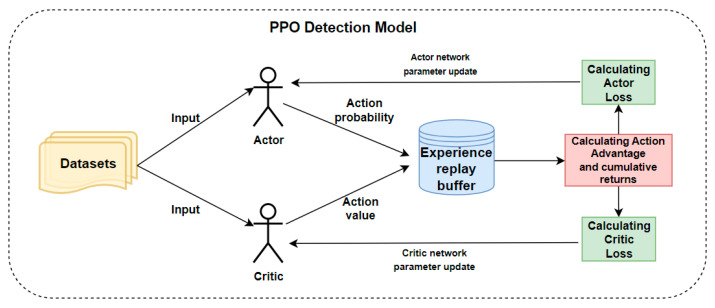
Diagrammatic representation of the working process of the PPO detection model.

After the model is constructed, it is trained using pre-processed datasets until a high level of accuracy is achieved. The working principle of the PPO malicious traffic detection model is illustrated in Figure 5 and Table 4.

## 4. Experimental Process and Results

### 4.1. Experimental Environment

The computer operating system used in this experiment is Windows 10, the CPU is i5-7300, RAM is 32G, the development environment is Python3.8.5 with Pycharm, and the Pytorch framework is used to construct the PPO detection model.

### 4.2. Selection of Indicators for Model Evaluation

The judgment in this study is based on the evaluation of the model in terms of whether it successfully identifies malicious traffic, which is a classification problem. Accuracy and F1 score [35] are commonly used metrics to evaluate model performance. Accuracy is one of the most intuitive evaluation metrics, representing the ratio of correctly predicted samples to the total number of samples. Therefore, it provides an intuitive measure that allows people to understand the overall performance of the model quickly. The F1 score combines the model’s precision and recall to provide a more accurate picture of the model’s performance on a small number of categories, taking into account the model’s accuracy and completeness. Therefore, this study uses accuracy and F1 score as the evaluation metrics of the model. The formulas for calculating accuracy and F1 score are as follows:

(9) $$Accuracy=\frac{TP+TN}{TP+TN+FP+FN}$$

(10) $$F1\mathrm{score}=2^{*}\frac{{\mathrm{Precision}}^{*}\mathrm{Recall}}{\mathrm{Precision}+\mathrm{Recall}}$$

Here, *TP* represents the number of true positive samples, which are positive samples predicted as positive by the model; *TN* represents the number of true negative samples, which are positive samples predicted as negative; *FP* represents the number of false positive samples, which are negative samples predicted as positive; and *FN* represents the number of false negative samples, which are negative samples predicted as negative.

The precision and recall mentioned in the F1 score formula refer to precision and recall, which are calculated as follows:

(11) $$\mathrm{Precision}=\frac{\mathrm{TP}}{\mathrm{TP}+\mathrm{FP}}$$

(12) $$\mathrm{Recall}=\frac{\mathrm{TP}}{\mathrm{TP}+\mathrm{FN}}$$

### 4.3. Analysis of Experimental Results

**Figure 6:**
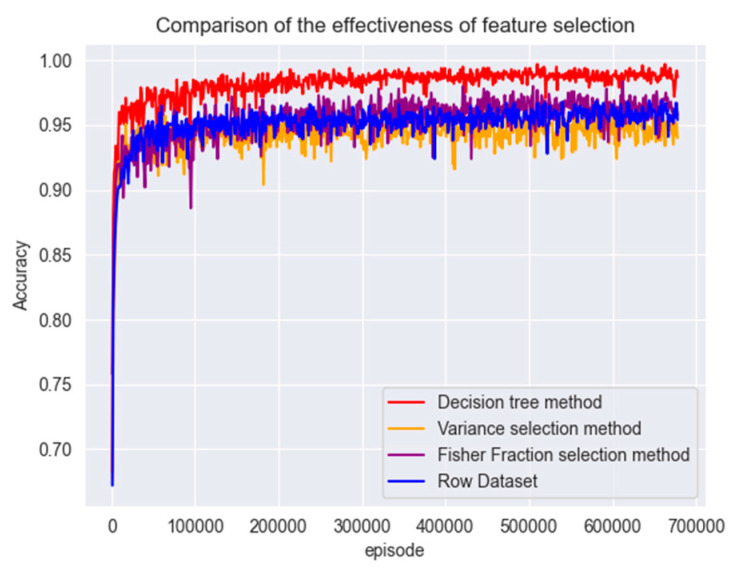
Comparison of feature selection accuracy.

**Figure 7:**
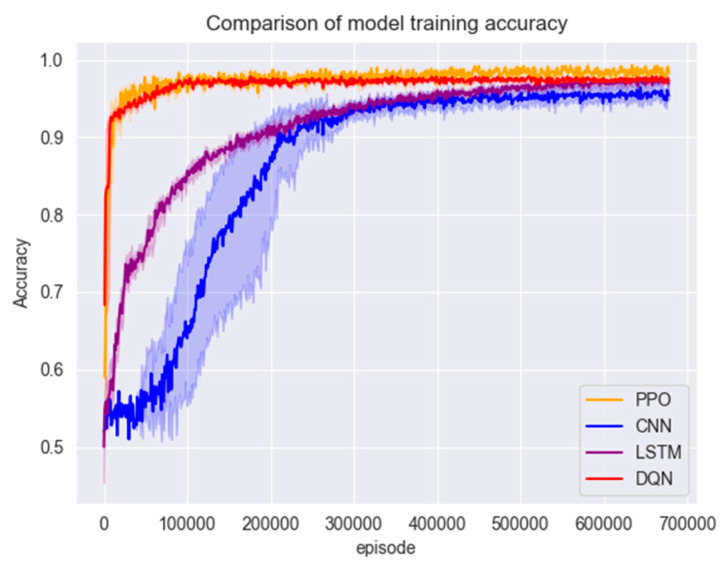
Model training set accuracy.

**Figure 8:**
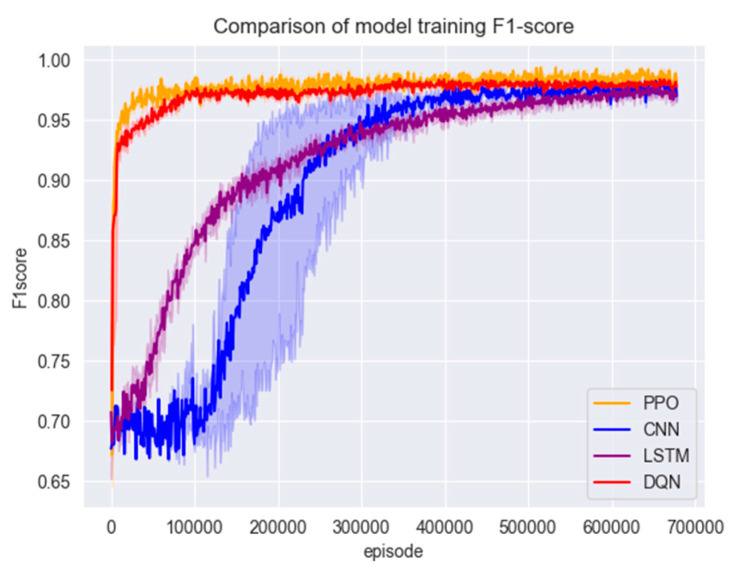
Model training set F1 score.

**Figure 9:**
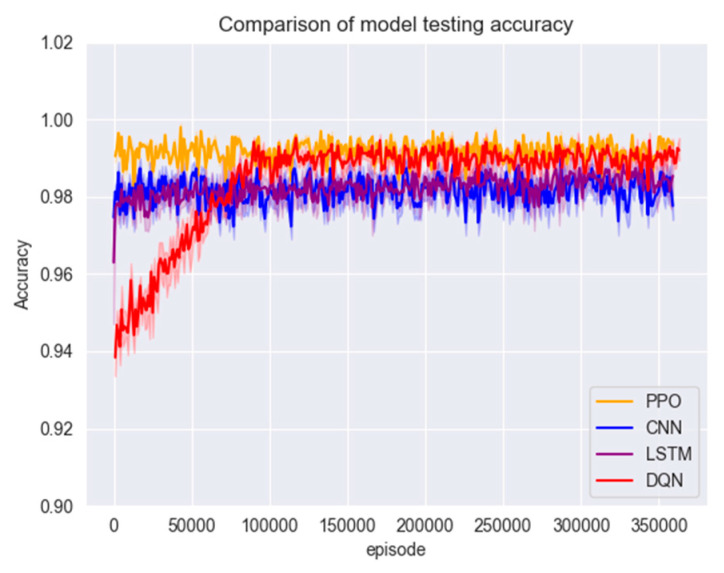
Model testing set accuracy.

**Figure 10:**
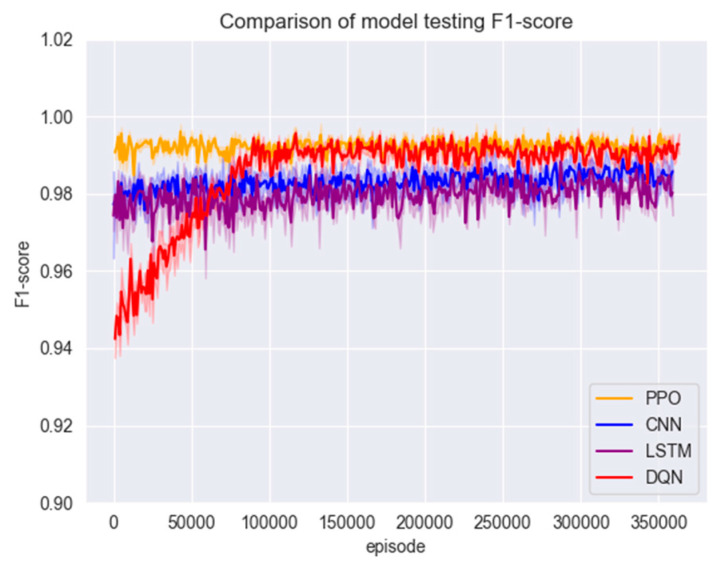
Model testing set F1 score.

**Figure 11:**
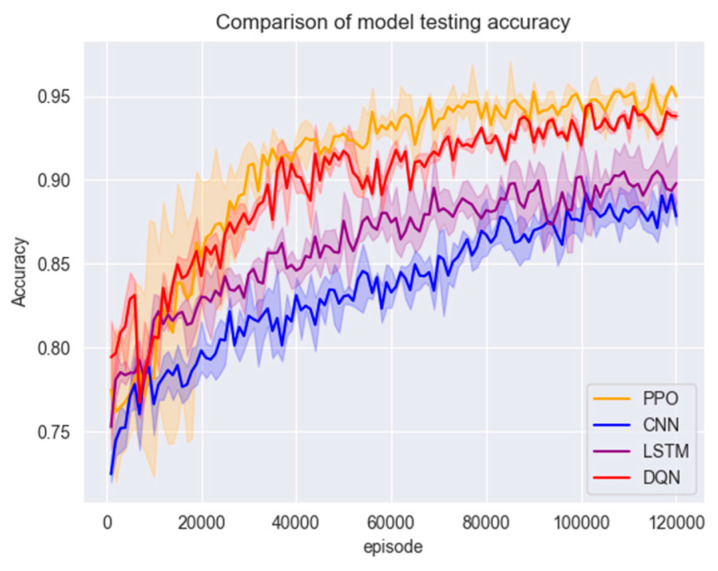
Comparison of model accuracy on random data.

**Figure 12:**
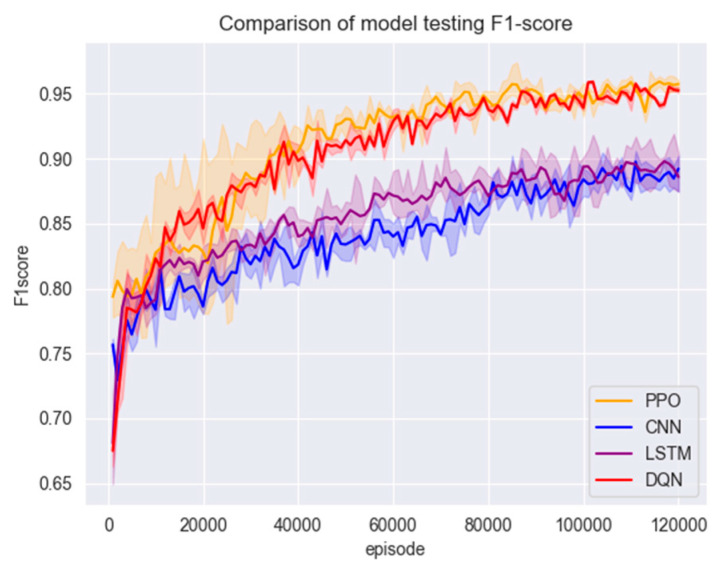
Comparison of model F1 scores on random data.

In this section, we will conduct three sets of controlled experiments to test the performance of the PPO algorithm model: First, train the model using a dataset that has undergone simple pre-processing and another dataset that has undergone feature selection, and analyse the effectiveness of the feature selection by comparing the results. Subsequently, the PPO, CNN [36], LSTM [37] and DQN [38] detection models will be trained and tested using the pre-processed CIC-IDS2017 dataset. Compare the convergence during training and the accuracy on the test set of the three models. Finally, the performance of the three models is validated by integrating CIC-IDS2017, CIC-Dos2017, CSE-CIC-IDS2018, and CIC-DDos2019 by labels and undergoing the same pre-processing. The performance of the three models is verified by randomly selecting some of the data to test the models’ ability to detect unfamiliar datasets. To mitigate randomness, all experiments in this section were repeated multiple times. The solid lines in Figure 6, Figure 7, Figure 8, Figure 9, Figure 10, Figure 11 and Figure 12 represent the average of multiple results from the same experiment. In contrast, the shaded areas represent the range of the maximum and minimum values of the experimental results.

#### 4.3.1. Comparison of Feature Selection Validity

In this section of the experiment, the PPO detection model is first trained using a dataset that undergoes simple data processing without feature selection; then, the decision tree classification algorithm is used to classify the dataset using the information entropy as the criterion for feature splitting to predict the classification, compute the importance score of each feature in the classification task, screen out the features with more significant information gains for retention, and use the new feature combination to model the training. Subsequently, feature selection was performed on the dataset using Fisher score and variance selection methods. The selected features were retained for model training. The obtained results are shown in Figure 6.

From Figure 6 it can be concluded that the decision tree classifier gave better results than the other two methods. Due to the complexity of the dataset, the Fisher Score method ignores the interactions and dependencies between features. This can lead to the selection of feature sets that contain redundant information. Entropy-based decision tree methods, on the other hand, make the dataset more regular and deterministic. Deep learning algorithms can learn these regularities faster and there is some improvement in the speed of convergence. After feature selection, the overall accuracy of the model is improved by 3.08%.

#### 4.3.2. Comparison of Model Performance

In this section of the experiment, the models are first trained using the training set in Section 3.1, and the changes in accuracy and F1 scores during the training of the three models are shown in Figure 7 and Figure 8.

Figure 7 and Figure 8 show that the PPO detection model exhibited a rapid convergence in the early stages of training, and the convergence process was highly stable. The model reached a relatively ideal state in a short period, which can be attributed to the PPO algorithm’s control over the magnitude of policy updates by introducing a truncated advantage function and approximate policy updates. This enhances the algorithm’s stability. Additionally, the presence of the experience replay buffer in the PPO algorithm enables higher sample utilisation efficiency and training efficiency.

Compared to traditional policy gradient methods, the PPO algorithm often converges faster and learns superior policies. Due to the need for feature extraction through convolutional and pooling layers, CNNs possess local connectivity and weight-sharing characteristics. This implies that in the initial stages, CNNs need to learn a large number of parameters and gradually adjust these parameters during training. This may result in the slower convergence of CNNs in the early stages of the training set. Although the DQN model initially showed good performance, the DQN algorithm tends to conduct more exploration, preventing the model from converging to an optimal state. Ultimately, the PPO algorithm outperforms the other three comparative models regarding accuracy, F1 score, and convergence speed.

After training with a large number of data samples, all four models converged to a better state; the test set was put into the models for testing to compare their accuracy and F1 scores; the results are shown in Figure 8 and Figure 9.

From Figure 9 and Figure 10, although the accuracy of all models reached a high level, the accuracy of the PPO algorithm model was still slightly higher than that of the other three models. Due to the presence of a clipping factor in the PPO algorithm, it ensures that the PPO algorithm does not encounter gradient explosion or vanishing issues. This allowed the model to converge to a very stable state during training, making the PPO algorithm model superior in stability compared to the CNN, LSTM, and DQN algorithm models. This can be verified from the F1 scores of the three models. The F1 score of the PPO algorithm model is significantly higher than those of the comparison models and has very little fluctuation, remaining within a stable range. In contrast, the F1 score curves of the other three models fluctuate significantly. Furthermore, the DQN model exhibited the worst performance in the early stages of testing. (See Figure 9 and Figure 10). The model test set data are shown in Table 5.

#### 4.3.3. Comparison of Performance on Other Datasets

To test the performance of the model on other unfamiliar datasets, four datasets, CIC-IDS2017, CIC-Dos2017, CSE-CIC-IDS2018 and CIC-DDos2019, were merged for the operation in this experiment, and then random data from the merged datasets were used to compare the accuracy and stability of the model. The results are shown in Figure 11 and Figure 12 and Table 6.

**Table 6:** Comparison of experimental data.

	Accuracy	F1-Score
CNN	0.8364	0.8425
LSTM	0.8624	0.8566
DQN	0.8976	0.9043
PPO	**0.9089**	**0.9111**

The data for this experiment were obtained by merging four datasets based on their features. After pre-processing and feature selection, some of the data were randomly selected and used to test the trained models. The purpose was to evaluate whether different detection models could effectively detect malicious network traffic when confronted with unfamiliar datasets. Figure 12 shows that all models experienced a decrease in accuracy and F1 score in the early stages of detection compared to the test set. However, due to its unique experience pool mechanism, the PPO algorithm improved the utilisation efficiency of samples. Therefore, after a brief adaptation period, it quickly iterated on the parameters, achieving better detection results faster than the other models. In the end, the detection accuracy of the PPO algorithmic model can still be maintained at around 95%, while the accuracies of the CNN, LSTM and DQN models are almost all lower than that of the PPO model, which fully proves the robustness and generalisation ability of the PPO algorithmic detection model.

## 5. Discussion

This paper proposes a malicious traffic detection model based on a machine learning algorithm decision tree classifier and deep reinforcement learning PPO algorithm. After pre-processing the data, it was fed into a decision tree classifier based on information entropy for initial prediction. Feature selection was then performed based on feature importance scores. Using the optimal number of features, the dataset was used to train and test the PPO algorithm model and the comparative models. Experimental results have shown that compared to existing CNN and LSTM detection models, and the PPO detection model exhibits a significantly faster convergence speed. Due to the presence of the ε-greedy algorithm in the DQN algorithm, the DQN model exhibited excessive fluctuations in both the training and testing sets. Due to the truncation factor set in the PPO algorithm, it can effectively control the magnitude of policy updates, avoiding the problems of gradient explosion or vanishing gradients. Therefore, the PPO algorithm model maintains high stability during rapid convergence, and its final detection performance still outperforms the comparative models. In the face of unfamiliar datasets, although the detection effect is reduced in the early stage, the PPO algorithm has an experience pooling mechanism, which can significantly improve the efficiency of using the sample experience and can be quickly adjusted to adapt to the new data. It can achieve better detection results faster than CNN and LSTM detection models.

Compared to the CNN and LSTM, the PPO model can achieve higher accuracy and better stability. When faced with unfamiliar datasets, the parameters can be updated more quickly, allowing the model to achieve a better level of detection. Compared to the DQN algorithm, the PPO model is able to perform more stably while maintaining a higher accuracy rate. The DQN model, on the other hand, gets lower metrics in the beginning due to the greedy algorithm. This conclusion can be drawn from the data of the training and test sets. Overall, the PPO algorithm is able to perform better in detecting malicious traffic. And it is able to remain relatively stable, which is exactly what is needed to detect malicious traffic.

## 6. Conclusions

Compared with the traditional deep learning detection model, the PPO algorithm detection model has some improvement in accuracy and stability. However, since the deep reinforcement learning algorithm does not just use neural networks for traffic detection, there are also mechanisms such as experience pools and the calculation of rewards, so the model complexity is relatively more significant, occupying more time and space costs. In subsequent research, deep reinforcement learning algorithms with multiple intelligences can be considered to improve the model to achieve more efficient malicious traffic detection while maintaining stability and accuracy and further saving costs. Regarding feature selection, the decision tree classifier algorithm only performs simple feature screening based on feature importance scores, and more advanced feature selection methods can be considered in subsequent research to screen the optimal combination of features to train the model and further improve the detection efficiency. It is a very worthwhile direction of research to consider improvements in computational cost to achieve better results with less model complexity when performing feature engineering. Although the research in this paper still has some limitations, it provides a new idea. Hopefully, it can give some experience and insights for developing more effective malicious traffic detection models.

## Figures and Tables

**Table 1:** The CIC-IDS2017 dataset data types.

Benign	DoS	DDoS	Botnet
Bruteforce	Infiltration	Portscan	Webattack

**Table 2:** Features with values all equal to 0.

Bwd PSH Flags	Bwd URG Flags	Fwd Avg Bytes/Bulk
Fwd Avg Packets/Bulk	Bwd Avg Bytes/Bulk	Bwd Avg Packets/Bulk
Fwd Avg Bulk Rate	Bwd Avg Bulk Rate	

**Table 3:** Removed 25 dimensions with highly correlated features.

Subflow Bwd Packets	Idle Mean	Flow Packets/s
Flow Duration	Total Backward Packets	min_seg_size_forward
Fwd Packet Length Std	Fwd IAT Std	Flow IAT Std
Flow IAT Max	Subflow Fwd Packets	Fwd IAT Max
Idle Min	Total Fwd Packets	Fwd Header Length
Max Packet Length	Total Length of Bwd Packets	Bwd Packet Length Std
Fwd Packet Length Mean	Bwd Packet Length Max	Total Length of Fwd Packets
Bwd Packet Length Mean	Packet Length Mean	Avg Bwd Segment Size
Average Packet Size		

**Table 4:** Pseudocode of the PPO algorithm.

The main pseudocode of the PPO algorithm in this paper is as follows:
1: Initialize the parameters of the Actor network. 2: Initialize the parameters of the Critic network. 3: Initialize the experience replay buffer. 4: Interact with the environment to obtain the initial environmental state *s*_0_. 5: Based on the current state *s*_0_, input the Actor network to obtain the probabilities of all actions, and sample an action *a*_0_ according to these probabilities. 6: Input *s*_0_ into the Critic network to obtain the action value *V*(*s*_0_). 7: Execute action *a*_0_, observe the next state *s*_1_, and the reward *r*_1_. 8: Calculate the action advantage *A* and the cumulative discounted return *G* for the current state using the advantage function. 9: Store an experience in the experience replay buffer: (*s*_0_, *a*_0_, *r*_1_, *v*(*s*_0_), log*P*(*a*_0_|*s*_0_), *A*(*a*_0_, *s*_0_), *G*_0_). 10: Retrieve a batch of data from the experience replay buffer and calculate the loss functions for the Actor and Critic networks. 11: Update the networks through gradient descent training. 12: If the terminal state is reached, proceed to step 3; otherwise, proceed to step 5.

**Table 5:** Comparison of experimental data.

	Training Set		Test Set	
	Accuracy	F1-score	Accuracy	F1-score
CNN	0.8623	0.8937	0.9816	0.9830
LSTM	0.9119	0.9153	0.9823	0.9796
DQN	0.9684	0.9725	0.9843	0.9854
PPO	**0.9810**	**0.9798**	**0.9917**	**0.9923**

## Data Availability

The original contributions presented in the study are included in the article, further inquiries can be directed to the corresponding author.
